# Improving rice grain length through updating the *GS3* locus of an elite variety Kongyu 131

**DOI:** 10.1186/s12284-018-0217-2

**Published:** 2018-04-10

**Authors:** Jianzong Nan, Xiaomin Feng, Chen Wang, Xiaohui Zhang, Rongsheng Wang, Jiaxin Liu, Qingbo Yuan, Guoqiang Jiang, Shaoyang Lin

**Affiliations:** 1 State Key Laboratory of Plant Genomics and National Plant Gene Research Center, Institute of Genetics and Developmental Biology, Chinese Academy of Sciences, Beijing, 100101 China; 2 University of Chinese Academy of Sciences, Beijing, 100039 China

**Keywords:** Rice, Kongyu 131, *GS3*, SNP, HRM, Breeding

## Abstract

**Background:**

Traditional crop breeding has made significant achievement meet food needs worldwide. However, the way has some inevitable problems including time-consuming, laborious, low predictability and reproducibility. In this study, we updated the *GRAIN SIZE 3 (GS3)* locus to improve the grain length of a major cultivate variety of Kongyu 131 at Heilongjiang Province, the northernmost region of China. High-resolution melting (HRM) analysis is used for single nucleotide polymorphism (SNP) genotyping.

**Results:**

The improved line introgressed about 117 kb segment including *gs3* allele from donor GKBR by using five SNP markers designed within and without *GS3* locus, and the background recovery ratio of the recurrent parent genome is about 99.55% that are detected by 219 SNP markers evenly distributed on the 12 chromosomes. The field trial indicates that grain length, 100-grain weight and total grain weight per plant of the improved line raised by 12.05%, 16.30% and 4.47%, respectively, compared with Kongyu 131.

**Conclusions:**

This result demonstrates that update the *GS3* locus is a feasible and efficient and accurate way can be applied to improve grain size of rice.

## Background

It is a great challenge enhancing production of crops to supply the increasing demand continuously; on the one hand, the arable land is decreasing with the development of urbanization, on the other hand, the production of crop is influenced inevitably by some environment factors of global warming more and more (Takeda and Matsuoka 2008). Some investigations indicate that total production of crops has growing slowly at some district, even decreased at other area (Ray et al. 2012). Therefore, it is an urgent challenge to supply the demand of accumulative population by increasing crop productivity.

Rice is a staple crop cultivated extensively, and it supply over half diets of population all around the world. Also rice is a model monocotyledon with a small genome and complete genome sequence and a large number of useful resources. It is considered that the production of per plant determined by panicle numbers per plant, grain numbers per panicle, grain weight and filling (Sakamoto and Matsuoka 2008; Xing and Zhang 2010). Grain weight is a vital factor influences production of rice; therefore, it is a potential way to improve production of rice by updating the locus associated with grain weight. And also the long-grain shape has been a new favored quality trait in the breeding of *japonica* rice in recent years (Haixiang and Qian 2017). Many grain size related genes in rice were identified in the past decade (Huang et al. 2013; Miura et al. 2011; Wang and Li 2011; Xing and Zhang 2010; Zuo and Li 2013). *GS3* is the first identified and molecularly characterized QTL controls grain size (Fan et al. 2006; Mao et al. 2010).

Traditional breeding almost depends on field selection by experience of breeders to improve crop variety. However, the selection has many inevitable defects including time-consuming and laborious, low predictability and reproducibility. Using markers, linkage with the target gene on the basis of DNA sequence, has greatly facilitate accurate and diminish time cost of selection, namely marker-assisted selection (MAS) (Knapp 1998). However, unavoidable recombination limited precision of selection between target gene and markers (Andersen and Lubberstedt 2003). Meanwhile, traditional breeding selection with MAS has no consideration of whole genome elimination of non-target fragments except for selection target phenotype. Therefore, it is still uncertain that the effects of non-target segments and reserve background genome of recurrent parent at the most extent.

The concept of design breeding was proposed by two scientists from Israeli (Peleman and van der Voort 2003). Now, it is possible that breeders can improve crop combining or incorporating beneficial genes using a great amount of genome and functional gene information with development of sequencing and molecular method technology (Huang et al. 2013; James et al. 2003; Miura et al. 2011; Sakamoto and Matsuoka 2008; Wang and Li 2008, 2011; Xing and Zhang 2010; Zhou et al. 2013; Zuo and Li 2013).

Kongyu 131 is a small grain variety, which once was a major cultivar in the Heilongjiang province of northeast China. This variety belong to a high-latitude *japonica*, which has early maturity, strong tillering strength and resist lodging, and average production about 7684.5 kg per hectare. In 2005, the planting area of Kongyu 131 reached 770,000 ha, accounting for more than half of the total rice planting area. And the planting area of Kongyu 131 reached more than 560,000 ha from 2006 to 2011. It is estimated that the planting area of Kongyu 131 still have least 400,000 ha last few years.

To improve the grain size of the elite variety Kongyu 131, we carried out QTL analysis with grain size and detected desirable allele around the *GS3* locus. Therefore we used the Update approach (Feng et al. 2017) to improve the grain size of a major cultivates variety of *japonica* Kongyu131. We developed SNP markers between Kongyu 131 and GKBR based on SNPs evenly distributed on rice 12 chromosomes. And also we designed five SNP markers at *GS3* locus (SNP3) including its upstream (SNP1 and SNP2) and downstream (SNP4 and SNP5) on the basis of sequence difference between Kongyu 131 and GKBR. SNP markers were identified by using HRM analysis (Wittwer et al. 2003). Finally, we selected a line, named improved line, which segment of *gs3* allele about 117 kb is from donor GKBR and the background recovery ratio of Kongyu 131’s about 99.55%. The field trial indicates that grain length and 100-grain weight of the improved line significantly heightened compared with Kongyu 131, and total grain weight per plant also increased. This result demonstrates that the Update approach is an efficient and accurate way can be utilized to improve grain size of rice varieties.

## Results

### QTL analysis detected a grain length related locus nearby *GS3*

**Fig. 1:**
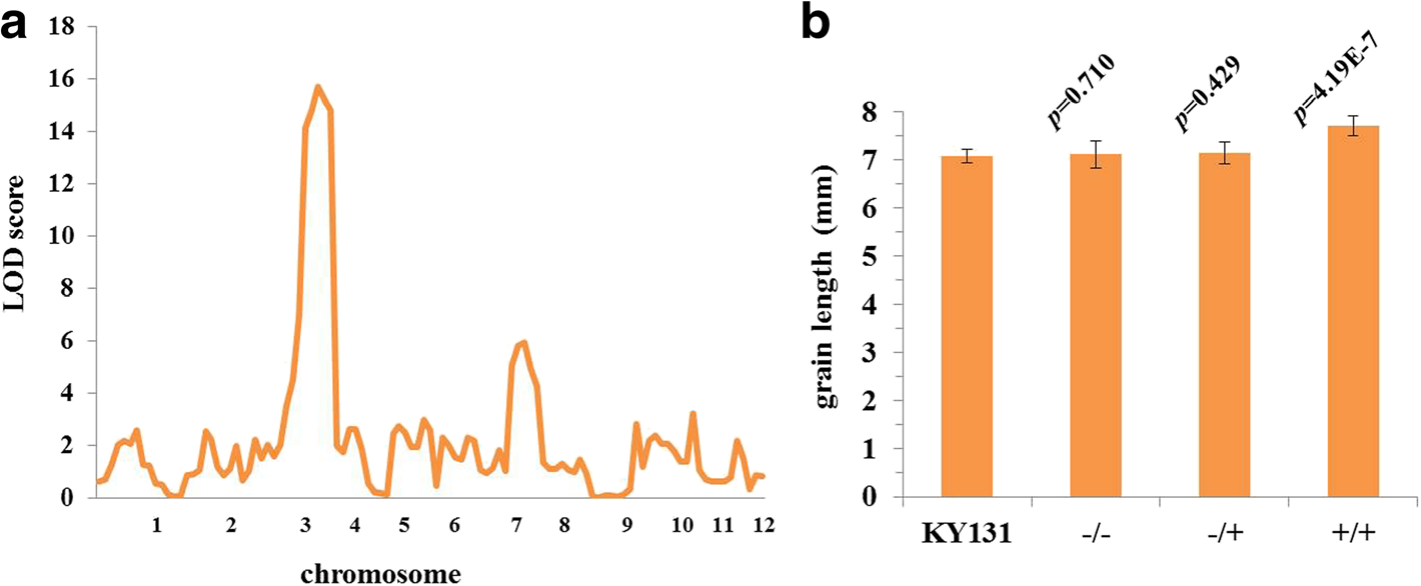
QTL analysis detected a locus related grain length nearby the *GS3* locus. **a** QTL analysis using F_2_ population with 136 individuals and 123 SNP markers. **b** the grain length is significantly increased with the donor GKBR allele at the *GS3* locus. *n* = 10. The values represent the mean ± s.d. *p* values from the student’s *t*-test of the plant with different genotype at *GS3* locus against Kongyu131 is indicated. KY131 is the abbreviation of Kongyu 131

To detect allele can increase Kongyu 131’s grain length, we measured grain length and analyzed genotype with an F_2_ populations constructed by Kongyu 131 and GKBR, and made QTL analysis according to Mapmaker/QTL 1.1b (Lincoln et al. 1993). We detected a QTL related to grain length on the chromosome 3 **(**Fig. 1a**)**, nearby *GS3* locus, presumably this QTL is *GS3* locus. And also we noticed another QTL on the chromosome 7, but we found this QTL from donor’s allele not only related with grain length but also delay heading date. Given the delayed heading date cannot fully mature in Heilongjiang, we only introgressed the QTL on the chromosome 3 in this study.

### Kongyu 131 with small grain allele and GKBR large grain allele at *GS3* locus

**Fig. 2:**
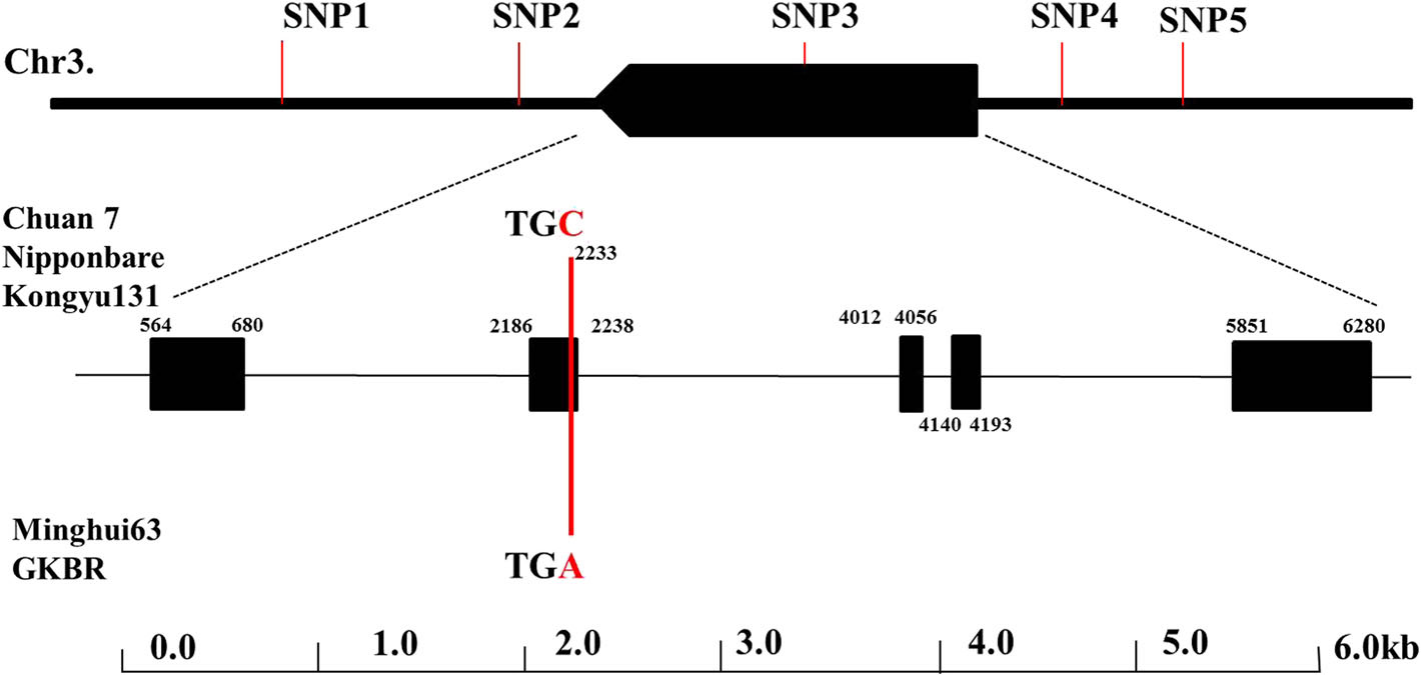
*GS3* sequence comparison and SNP markers used to select for *gs3*. Kongyu 131 and GKBR has a base variance with the red indicates at the second exon as same as difference between Chuan 7 and Minghui 63 from which the *GS3* gene was first cloned

To identify whether donor GKBR has large grain allele at *GS3* locus or not, we compared the coding DNA sequence (CDS) of *GS3* gene between Kongyu 131 and GKBR using software of DNAMAN. We found a base difference at the second exon between Kongyu 131 and GKBR **(**Fig. 2**)**. This variance at *GS3* locus is same as the difference between a small grain cultivar, Chuan 7, and a large grain cultivar Minghui 63, which are two parents used to clone the *GS3* for the first time. And also the researchers demonstrated that the variance of *GS3* lead to premature termination of transcription in the large grain varieties (Fan et al. 2006; Mao et al. 2010). Therefore, we supposed that Kongyu 131 is the small grain allele and GKBR is the large grain allele at *GS3* locus.

### Five SNP markers used to select for *gs3* allele from donor GKBR

To select for *gs3* allele from donor GKBR accurately, we developed a SNP marker, named SNP3 **(**Fig. 2**)**, located within *GS3* gene. And also we designed other four SNP markers- SNP1, SNP2, SNP4, SNP5 **(**Fig. 2**)**- located at upstream and downstream of *GS3* gene, respectively, the physical distance about 1 Mega base pair (Mbp) between SNP1 and SNP5. These four markers mainly used to eliminate the drag of non-targeted segment.

### Genotype of an individual incorporated around 117 kb at *GS3* locus from donor GKBR

**Fig. 3:**
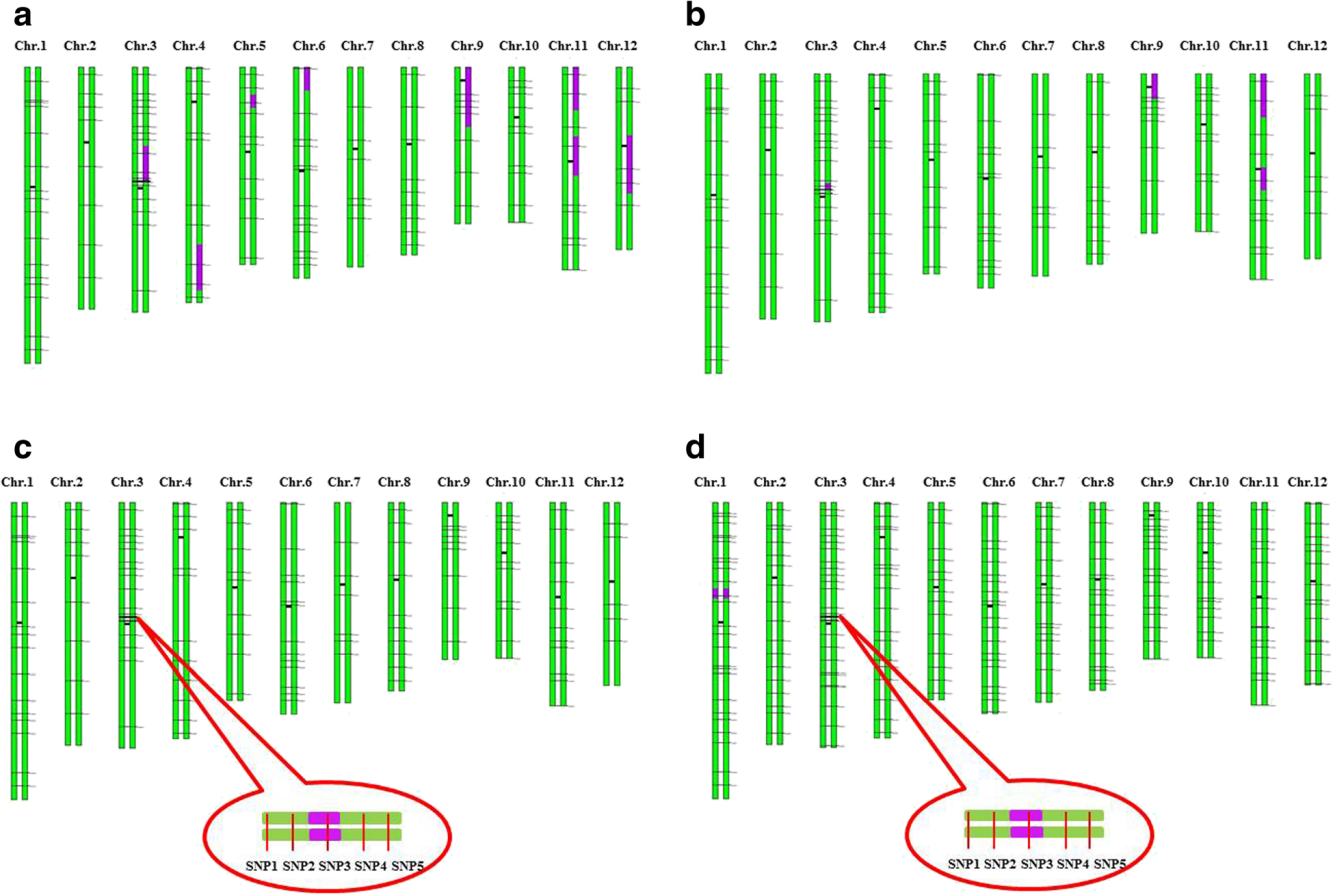
Graphical Genotype (GGT) of selected individuals or lines. **a** GGT of the first selected individual BC3F1–1 had recombination between SNP3 and SNP5. **b** GGT of the selected individual BC3F2–2 had recombination between SNP3 and SNP4. **c** GGT of the selected individual BC3F3–3 with recombination between SNP3 and SNP2. **d** GGT of the improved line BC3F4–4 with the highest genetic background recovery ratio. The green type means the chromosomes of Kongyu 131, and the purple represents the fragments from GKBR, the horizontal black lines indicate the physical position of SNP markers

To acquire individuals incorporated the smallest segment of *gs3* from donor GKBR. First, we selected 25 individuals with heterozygote genotype at SNP3 from the BC_3_F_1_ population. And we found 8 of 25 individuals had recombination between SNP1 and SNP5. An individual was selected with highest genetic background recovery ratio from the 8 individuals using 134 SNP markers, named BC3F1–1 **(**Fig. 3a**)**, which had recombination between SNP3 and SNP5. Second, we selected an individual, named BC3F2–2 **(**Fig. 3b**)**, which had recombination between SNP3 and SNP4, from the progeny of BC3F1–1. Third, to get individuals had recombination between SNP3 and SNP1 or SNP2, we further selected from 400 progeny of BC3F2–2. Finally, we selected an individual with recombination between SNP3 and SNP2. We dubbed the selected individual BC3F3–3 **(**Fig. 3c**)**, which incorporated about 117 kb homozygote segment from donor GKBR at *GS3* locus.

### The improved line with genetic background recovery ratio 99.55%

To get the improved line with highest genetic background recovery ratio, we selected an individual named BC3F4–4 **(**Fig. 3d**)**, which genetic background recovery ratio of Kongyu 131 is about 99.55%, using 219 SNP markers evenly distributed on 12 chromosomes from the progeny of BC3F3–3. And also we found a non-target segment at chromosome 1 **(**Fig. 3d**)** according to those increased 85 markers, which had not been identified at BC3F1–1, BC3F2–2 and BC3F3–3.

### Grain length and weight of the improved line significantly increased

**Fig. 4:**
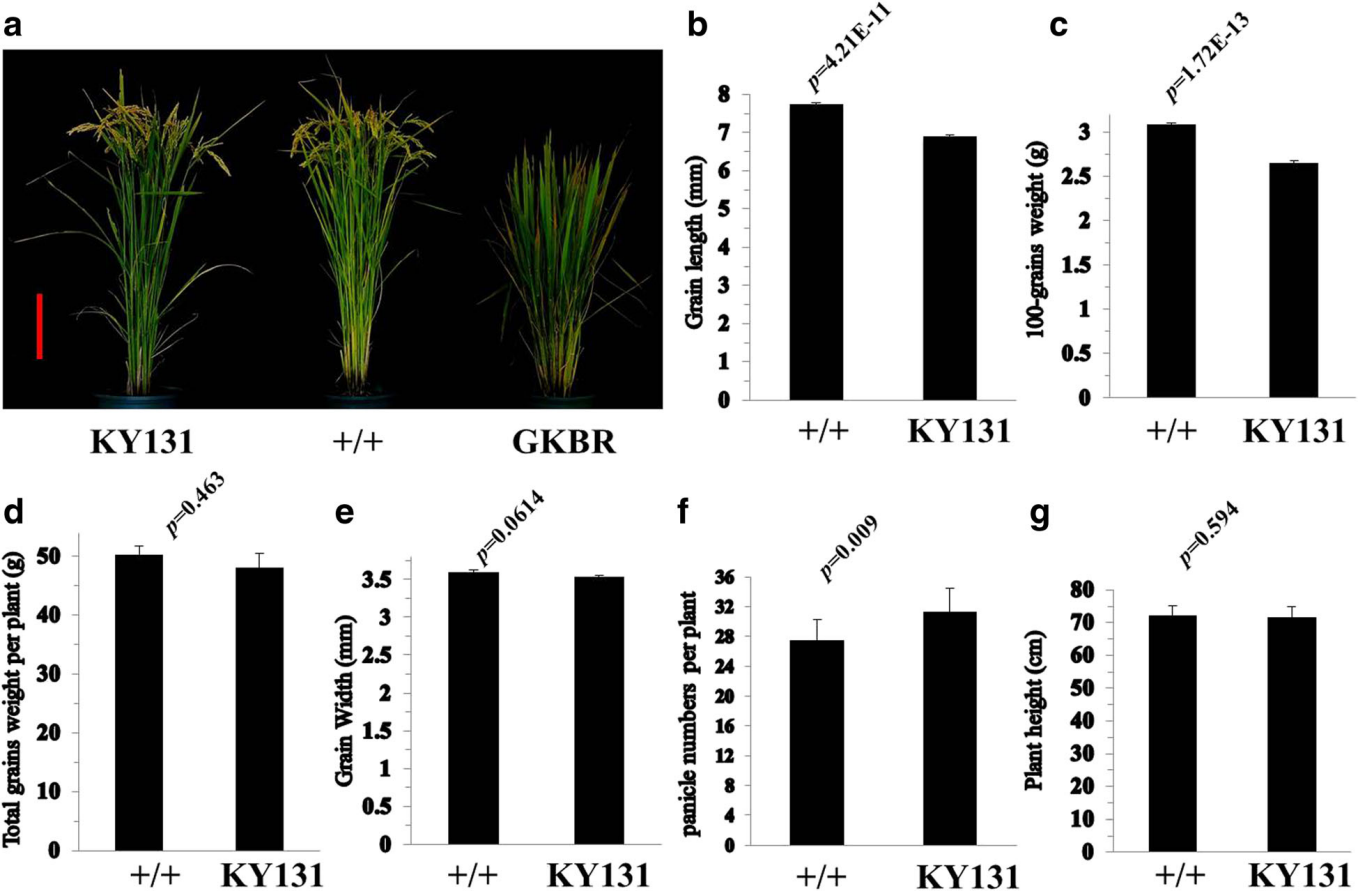
Grain length and 100-grain weight significantly increased of the improved line compared with Kongyu 131. **a** Plant type of recurrent parent Kongyu 131, the improved line and the donor GKBR. Scale bar, 30 cm. **b**-**g** Comparison of yield-related traits of Kongyu 131 and the improved line. For b-g: *n* = 10. The value in b-g represent the mean ± s.d. *p* values from the student’s *t*-test of the improved line against Kongyu131 is indicated. KY131 is the abbreviation of Kongyu 131

To verify *gs3’*s function of the improved line, we cultivated Kongyu 131 and the improved line 96 individuals with three replications at Heilongjiang, respectively. According to general management of trial plot, we investigated traits including grain length, grain width, 100-grain weight, plant height and total grain weight and so on (Table 1). The improved lines’ grain length **(**Fig. 4b**,** Fig. 6a**)** and 100-grain weight **(**Fig. 4c**)** increased significantly compared with Kongyu 131, and total grain weight increased non-significant **(**Fig. 4d**)**. We also found the number of panicle per plant of the improved lines decreased significantly (Fig. 4f). In some sense, this result can explain the reason why the improved lines’ total grain weight increased non-significant while 100-grain weight increased significantly. The result manifests that the improved lines’ grain length and 100-grain weight increased significantly compared with Kongyu 131. Thus, the method is efficient and accurate update the *GS3* locus of Kongyu 131.

**Table 1:** Agronomic performance of Kongyu 131 and its improved line BC4F4–4 in 2016 and 2017

Traits	2016		2017	
	BC3F4–4	KY 131	BC3F4–4	KY 131
GL(mm)	7.73 ± 0.14*	6.90 ± 0.11	7.90 ± 0.13*	6.94 ± 0.09
GW(mm)	3.59 ± 0.08	3.53 ± 0.05	3.47 ± 0.11	3.44 ± 0.03
HGW(g)	3.08 ± 0.04*	2.65 ± 0.05	2.98 ± 0.05*	2.73 ± 0.06
TYP(g) ^a^	50.27 ± 4.81	48.11 ± 7.7	48.82 ± 6.45	48.39 ± 5.87
TYP(g) ^b^	55.96 ± 11.16*	40.82 ± 4.31	–	–
TYP(g) ^c^	–	–	48.52 ± 8.26	48.06 ± 3.79
PNP	27.4 ± 2.87*	31.3 ± 3.16	24.13 ± 1.73*	34 ± 3.38
GNP	125.8 ± 10.51	116.8 ± 23.5	126.5 ± 14.13	116.38 ± 8.42
PH(cm)	72.2 ± 2.82	71.45 ± 1.06	72.63 ± 0.58*	67.60 ± 1.75
PL(cm)	17.7 ± 1.22	17.58 ± 1.74	16.28 ± 1.35	16.06 ± 0.63
DTH	107.25 ± 2.06	107.25 ± 2.63	102.50 ± 1.64	102.83 ± 2.14

Data presented as the means with standard deviations were obtained from plants in a randomized complete block design with three replications under natural conditions at Jiamusi in 2016 and 2017. The planting density was 30 cm × 20 cm and one plant per hill

*GL* Grain length, *GW* Grain width, *HGW* Hundred grain weight, *TYP* Total grain yield per plant, *PNP* panicle number per plant, *GNP* grain number of main panicle, *PH* plant height, *PL* main panicle length, *DTH* Days to heading

*represents significance at *p* ≤ 0.05 based on Student’s *t*-tests, *n* = 10

^a^The planting density was 30 cm × 20 cm and one plant per hill

^b^The planting density is 30 cm × 20 cm and three to four plants per hill

^c^The planting density is 30 cm × 14 cm and three to four plants per hill; − indicates no data

### The allele of GKBR at *GS3* locus can increase Kongyu 131’s grain length according to QTL analysis

**Fig. 5:**
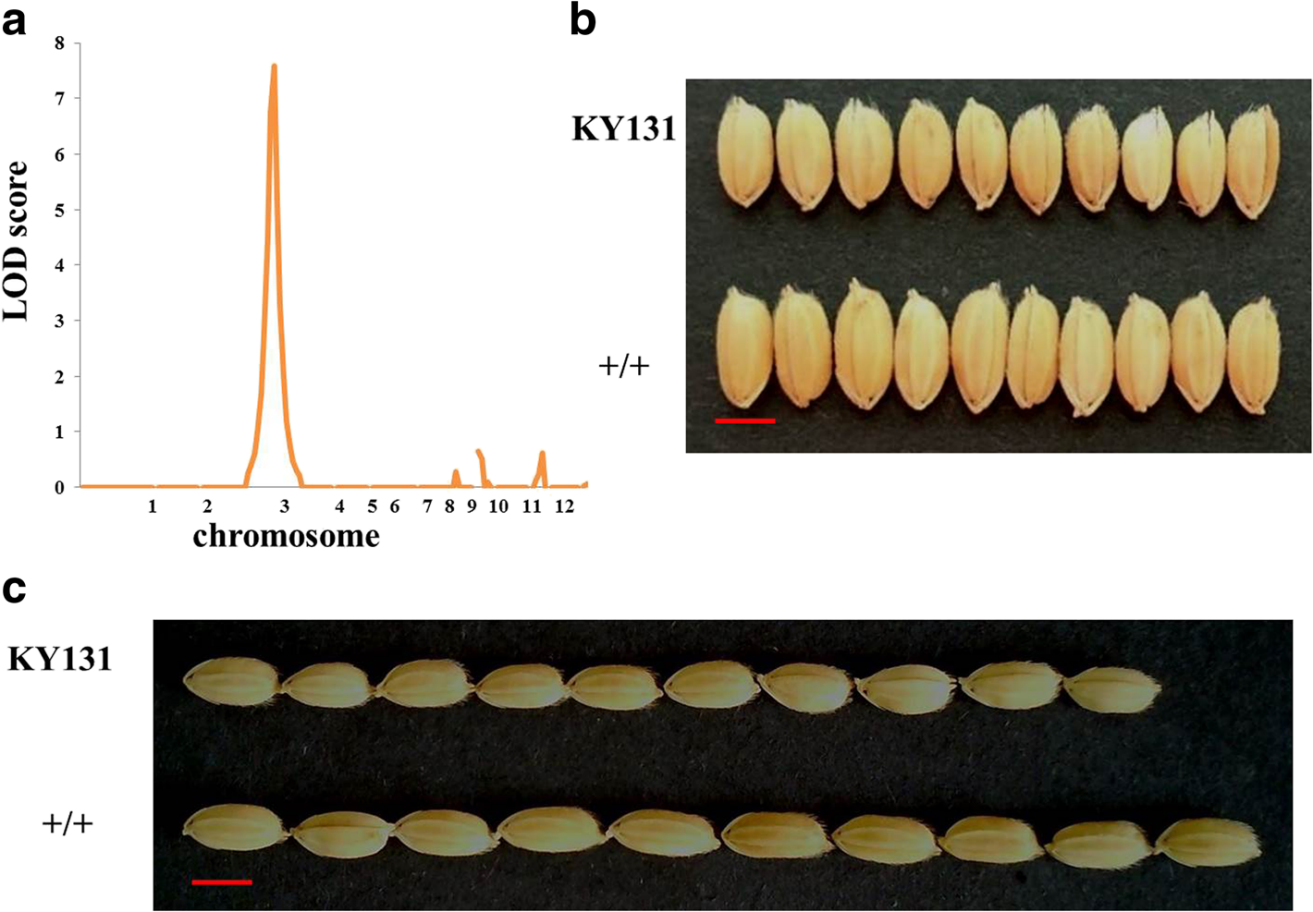
QTL analysis confirmed that *gs3* allele from donor GKBR increased grain length significantly. **a** QTL analysis using BC_3_F_2_ population with 46 lines and 138 SNP markers. **b, c** the grain length is significantly increased with the donor GKBR allele at *GS3* locus. Scale bar, 5 mm. KY131 is the abbreviation of Kongyu 131

To further confirm the allele of GKBR at *GS3* locus can increase Kongyu 131’s grain length, we made QTL analysis using BC_3_F_2_ population constructed by Kongyu 131 and GKBR. We detected a QTL on chromosome 3 and the largest LOD score at the SNP3 **(**Fig. 5a**)**, presumably this QTL is *GS3* locus. Therefore, we confirmed that the allele of GKBR at *GS3* can increase Kongyu 131’s grain length **(**Figs. 5c**,** 6a**)**.

**Fig. 6:**
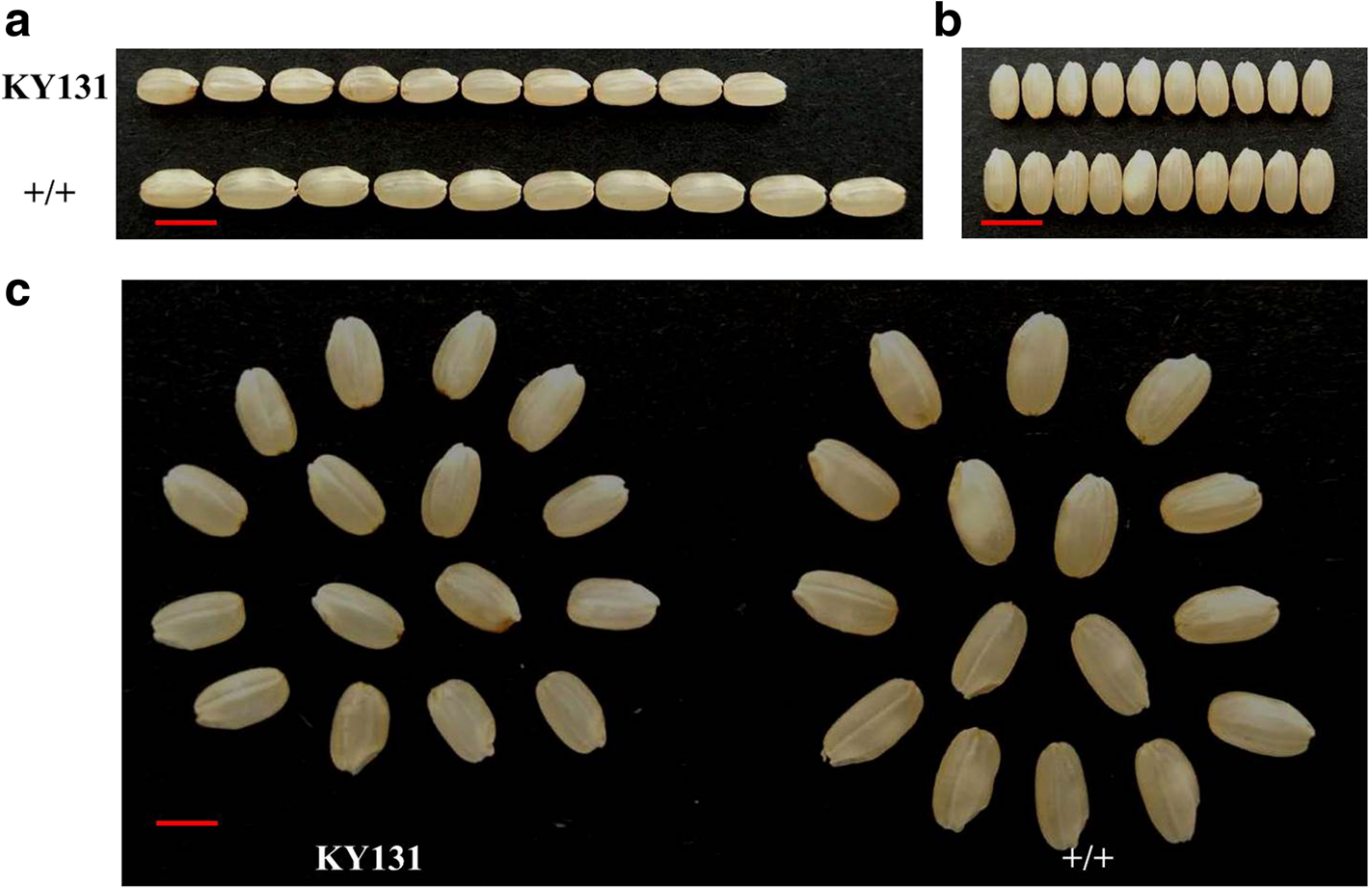
Grain comparison between the improved line and Kongyu 131. **a** Grain length comparison. **b** Grain width comparison. **c** Grain appearance comparison between the improved line BC3F4–4 and Kongyu 131. Scale bar, 5 mm. KY131 is the abbreviation of Kongyu 131

## Discussion

In this study, we applied the new approach updated the *GS3* locus of Kongyu 131, and verified grain length and 100-grain weight increased significantly of the improved line compared with Kongyu 131. So we convinced that the Update method is an accurate and efficient method to improve rice variety. First of all, the selection of target individual using the method is according to genotyping with SNP markers. Thus, the method overcomes the defects of time-consuming of traditional field selection by breeders’ experience. Second, the selection of individual incorporated target gene by SNP marker within gene, so, this selection no concerns of omit target gene. Third, the selection process including not only forward selection but also background elimination. According to background elimination, on the one hand, it eliminate the effects of non-target segment, on the other hand, it reserves the background genome of recurrent parent at the most extent. More importantly, the way can update causal gene as soon as we find defects of the improved line. On the basis of above results, we convinced that the approach overcomes the defects of time-consuming, low predictability and reproducibility of traditional breeding. Thus, it is predictable that the way will be applied to improve crop to satisfy the increasing demand as an accurate and controlled and efficient method.

Meanwhile, we noticed that the total grain weight per plant of the improved line increased nonsiginificantly compared with Kongyu 131 when the planting density is 30 cm × 20 cm and one plant per hill (Table 1). Also we find the total grain weight of the improved line increased siginificantly compared with Kongyu 131 when the planting density is 30 cm × 20 cm and three to four plants per hill (Table 1). More importantly, the total grian weight of the improved line when the planting density is 30 cm × 20 cm and three to four plants per hill increased more than when the planting density is 30 cm × 14 cm and three to four plants per hill. Thus, we believed that the total grain weight can increased significantly according to appropriate planting density. Next, we will verify the adapted planting density of the improved line by field experiment.

### Traditional breeding with MAS and update breeding

The selection process of traditional breeding almost depends on field selection by experience of breeders. However, this selection has many inevitable defects including time-consuming, low predictability and reproducibility. Although marker-assisted selection (MAS) using markers on the basis of DNA sequence has greatly facilitate the accurate and diminish the time-cost of selection, unavoidable recombination limited precision of selection between target gene and markers, which mostly is linkage with target gene. But the selection process of Update breeding used in this study has no concern of omit target gene by using marker within gene.

Meanwhile, we designed four SNP markers anchored upstream and downstream of target gene at the process of selection using the Update method; those four markers are used to eliminate the drag of non-target segments surrounding the target gene. This process cannot achieve by using MAS, no mention to field selection process.

The traditional breeding selection and MAS have no consideration of whole genome scanning, namely background elimination, except select for target phenotype. But the Update breeding selection including forward selection and background elimination scanning whole genome. According to background elimination it not only eliminates the effects of non-target segment but also reserves the background of recurrent parent at the most extent.

According to reserve the background of the recurrent parent, the method resolved the defect that the traditional breeding hard to reusing the genome information. On the basis of whole genome sequence information, we can continuously detect and update causal gene lead to defects of the cultivar improved by the Update breeding. According to this process, the beneficial allele will accumulated within recurrent parent, and this can become a reality that cultivars updated by successively improving in the future, as computer software update.

As above mentioned, we can eliminate the non-target segment at chromosome 1 **(**Fig. 3d**)** by backcross with Kongyu 131 as soon as we found defects influenced by it at the cultivate process. This process of improvement reserves the background of the recurrent parent at the most extent.

### Genome information and update breeding

We reckon that some elements must to be consideration to apply the Update breeding on the basis of above results and analysis. First, the prerequisite is the reliable and accurate genome sequence information. Second, also the thorough study of functional gene of many traits is a necessary foundation. Third, information management with high accuracy and efficient is vital necessity.

Many crops genome has sequenced or is sequencing with the development of sequencing technology. This information facilitates utilizing the Update breeding method in the future. However, there is much room to advance the accuracy of genome sequence information which will facilitate precision of whole genome elimination. And it is more efficient and accurate to update allele of specific trait using the Update breeding only when causal functional gene is definite. So it is vital to broad scope of identification and functional analysis of many genes. More importantly, management of information with accurate and efficient way is necessary to guarantee using that exactly.

With the development of exact genome sequence information and definite functional gene and high-efficiency information management, we hopefully believed that the Update breeding will be extensively applied to improve and update crops. And thus, it will satisfy the demand of increasing population all around the world.

## Conclusions

To improve the grain size of an elite variety Kongyu 131, we carried out QTL analysis and detected desirable allele from donor GKBR around the *GS3* locus. And we updated the grain size by introgressed the favorable allele from the donor using designed five SNP markers within and without *GS3.* Compared with Kongyu 131, grain length and 100-grain weight and total grain weight per plant of the improved line increased by 12.05%, 16.30% and 4.47%, respectively. This result demonstrates that update the *GS3* locus is a feasible and efficient and accurate way can be applied to improve grain size of rice.

## Methods

### Parents and populations

Kongyu 131, a small grain variety, used as the recurrent parent in this study. And the donor, GKBR, is an *indica* rice with longer grain while unable to be normal maturity in Heilongjiang province for inappropriate photoperiod and temperature conditions. GKBR was crossed with Kongyu 131 and the F1 was backcrossed with Kongyu 131 to produce a BC_3_F_1_ population including 137 lines and so on. The large allele *gs3* from GKBR was used in this study.

### Re-sequencing genome of two parents and sequence comparison of *GS3*

We acquired the genome sequence and SNPs of the two parents of Kongyu 131 and GKBR by next generation sequencing (NGS) using HiSeq2000. And we downloaded *GS3* sequence of Chuan 7 from database of NCBI (https://www.ncbi.nlm.nih.gov/), then made sequence comparison between Kongyu 131 and GKBR to verify the donor have large grain allele at *GS3* locus.

### The function of *GS3*

*GRAIN SIZE 3(GS3)* is the first molecularly characterized major QTL controls grain size and weight in rice. *GS3* was identified from the progeny derived from a cross between Minghui 63 with large grain and Chuan 7 with small grain, which show remarkable difference in grain length and weight. According to map-based clone, the researchers found *GS3* encodes a putative transmembrane protein containing a plant-specific organ size regulation (OSR) domain, a tumor necrosis factor receptor/nerve growth factor receptor (TNFR/NGFR) family cysteine-rich domain, and a von Willebrand factor type C (VWFC) domain (Fan et al. 2006; Mao et al. 2010; Zuo and Li 2014). The large grain variety, Minghui 63, has a base difference, which is adenine (A) compared with small grain variety, Chuan 7 with cytosine (C) at the second exon. The single nucleotide mutation detected at the *GS3* gene between these two different grain-length groups, which changed a cysteine codon (TGC) in the small-grain group to a termination codon (TGA) in the large-grain group. This nonsense mutation lead to premature termination of transcription at the long grain variety, indicating that long grain resulted from the loss of the function of the protein otherwise producing short grains (Fan et al. 2006).

### Development of SNP markers and genotyping

We designed more than 219 SNP markers evenly distributed on the whole genome between Kongyu131 and GKBR based on SNPs from the re-sequenced data. Those markers are used to scanning whole genome, namely background elimination. Also we developed five SNP markers (Table 2)within and without *GS3* locus used to forward selection. Those markers are confirmed by HRM analysis.

**Table 2:** Five SNP markers used to selection target chromosome segment

Markers	Chr.	Position	Forward primer	Reverse primer
SNP1	3	16,854,214	TGGTACACAGCATATCATGGAAC	CAGAAGTGTTATAACTACATATTTGC
SNP2	3	17,296,672	ATCTGCAACAAACAAGAGGATC	CTTGAGTTTCCACTCACAAACTTTTC
SNP3	3	17,369,402	GAAACAGCTGGCTGGCTTACTCTC	GATCCACGCAGCCTCCAGATGC
SNP4	3	17,413,766	TTAGGACATATCGGCGTGCGTTTA	CAGAGAAGCATCATTGAACGAACA
SNP5	3	17,874,647	ATGCAACCTTTTCTCCCTTCCTAT	TCTAAAGGTTAACTCAGTAAAATCCT

### Selection for the improved line

**Fig. 7:**
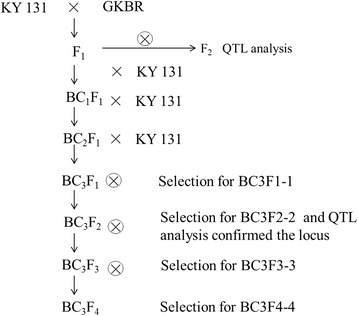
Procedure of selection for the improved line BC3F4–4

To gain the improved line incorporated the smallest segment at *GS3* locus from donor GKBR, and also have the highest genetic background recovery ratio, at the beginning of selection, we selected individuals with heterozygote genotype at SNP3 from BC_3_F_1_ population. And then, we used markers of SNP1-SNP5 select for individuals with recombination between SNP3 and SNP1 or SNP2 or SNP4 or SNP5. Namely, we selected individuals with recombination between SNP3 and its upstream and downstream at the same time. Finally, we selected an individual incorporated homozygote segment from donor GKBR at *GS3* locus, and also with the highest genetic background recovery ratio (Fig. 7).

### The field plot trial identify the *GS3*’s function of the improved line

To verify *GS3* function of the improved line, we cultivated Kongyu 131 and the improved line 96 individuals with three replications at Heilongjiang (130°57′E, 46°23′N), respectively. According to general management of field, we investigated traits including grain length, grain width, 100-grain weight, plant height and total grain weight.

## Abbreviations

GS3: Grain size 3
HRM: High-resolution melting
MAS: Marker-assisted selection
QTL: Quantitative trait loci
SNP: Single nucleotide polymorphism
